# Feasibility and usability of microinteraction ecological momentary assessment using a smartwatch in military personnel with a history of traumatic brain injury

**DOI:** 10.3389/fneur.2025.1564657

**Published:** 2025-04-14

**Authors:** Katrina Monti, Katie Williams, Brian Ivins, Jay Uomoto, Jami Skarda-Craft, Michael Dretsch

**Affiliations:** ^1^Traumatic Brain Injury Center of Excellence, Silver Spring, MD, United States; ^2^CICONIX LLC, Annapolis, MD, United States; ^3^Madigan Army Medical Center, Tacoma, WA, United States; ^4^General Dynamics Information Technology, Falls Church, VA, United States; ^5^Department of Psychiatry and Behavioral Sciences, University of Washington, Seattle, WA, United States

**Keywords:** microinteraction, ecological momentary assessment, experience sampling, smartwatch, military, traumatic brain injury

## Abstract

**Introduction:**

Microinteraction Ecological Momentary Assessment (miEMA) addresses the challenges of traditional self-report questionnaires by collecting data in real time. The purpose of this study was to examine the feasibility and usability of employing miEMA using a smartwatch in military service members undergoing traumatic brain injury (TBI) rehabilitation.

**Materials and methods:**

Twenty-eight United States active duty service members with a TBI history were recruited as patients from a military outpatient TBI rehabilitation center, enrolled in either a 2-week or 3-week study arm, and administered miEMA surveys via a custom smartwatch app. The 3-week arm participants were also concurrently receiving cognitive rehabilitation. Select constructs evaluated with miEMA included mood, fatigue, pain, headache, self-efficacy, and cognitive strategy use. Outcome measures of adherence were completion (percentage of questions answered out of questions delivered) and compliance (percentage of questions answered out of questions scheduled). The Mobile Health Application Usability Questionnaire (MAUQ) and System Usability Scale (SUS) assessed participants’ perceptions of smartwatch and app usability.

**Results:**

Completion and compliance rates were 80.1% and 77.4%, respectively. Mean participant completion and compliance were 81.1% ± 12.0% and 78.1% ± 13.0%, respectively. Mean participant completion increased to 87.7% ± 8.8% when using an embedded question retry mechanism. Mean participant survey set completion was 69.8% ± 18.3% during the early morning but remained steady during the late morning/early afternoon (85.7% ± 12.8%), afternoon (86.2% ± 12.6%), and late afternoon/evening (85.0% ± 14.7%). The mean overall item score for the MAUQ was 6.3 ± 1.1 out of 7. The mean SUS score was 89.0 ± 7.2 out of 100 and mean SUS percentile ranking was 96.4% ± 8.4%.

**Conclusion:**

Overall adherence was similar to previous studies in civilian populations. Participants rated the miEMA app and smartwatch as having high usability. These findings suggest that miEMA using a smartwatch for tracking symptoms and treatment strategy use is feasible in military service members with a TBI history, including those undergoing rehabilitation for cognitive difficulties.

## 1 Introduction

Persistent or variable symptoms occur in a subset of patients following mild traumatic brain injury (TBI) (1–3), and the ability to monitor symptom changes and treatment compliance is a critical component of TBI rehabilitation. Conventional approaches to assess and monitor symptoms rely on the use of self-report questionnaires which are often administered at multiple visits. Questionnaire selection may vary depending on the provider, symptomology, and treatment goals. Self-report questionnaires require patients to retrospectively recall and aggregate symptoms over a period of time, often over the previous 2 weeks or 30 days. This traditional approach has inherent challenges, specifically recall biases and questionnaire fatigue, which can significantly affect the quality and validity of self-reported data (4). Inaccurate or unreliable self-reports could lead to medically inappropriate or ineffective treatment decisions. The nature of traditional self-report questionnaires makes them inadequate for capturing evolving symptomology within real-world contexts over time. Military service members may be especially subject to questionnaire fatigue due to the many surveys they are asked to complete (5) (i.e., Climate Assessment surveys, military medical treatment facility [MTF] experience surveys, annual Periodic Health Assessments, and surveys related to the morale, family support, readiness, and health risks and behaviors across the Department of Defense). Therefore, approaches to assess and obtain more accurate and ecologically valid data for various symptoms, monitor treatment compliance, and assess other social, psychological, and behavioral factors related to TBI while minimizing survey fatigue are needed in military patient populations to optimize treatment intervention efforts.

Ecological Momentary Assessment (EMA), a term originally devised by Stone and Shiffman (6), is an *in-situ* experience sampling method that has demonstrated promise in addressing the previously stated challenges to inform just-in-time adaptive interventions (6–8). EMA has been used to assess symptoms, behaviors, and other factors related to substance abuse (9), suicidal ideations and relevant risk factors (10), post-traumatic stress disorder (11), and mood disorders (12). Standard EMA approaches allow for the collection of intensive longitudinal data through repeated assessments of variables and outcomes of interest that occur throughout a person’s daily life and within their natural settings. These data include timestamped momentary measures obtained while they are interacting with their work, community, and home environments (8). In this way, subjective experiences captured in real-time can account for within-day variations, avoid the pitfalls of retrospective recall, minimize survey fatigue, and optimize the ecological validity of responses (6). Standard EMA question sets may consist of dozens of questions, response options may be multiple choice, open-ended, or sliding scale, and questions may take 1–2 minutes (min) to answer (13, 14). EMA questions are usually delivered through a mobile phone or other personal digital device (13, 14). Despite the observed advantages of EMA compared to traditional questionnaires, interruption burden and intrusiveness remain important limitations and where participant engagement and data quality have been shown to reduce over time (15–18).

There is a growing body of evidence that the use of Microinteraction Ecological Momentary Assessment (miEMA) may be preferable to standard EMA because microinteractions have been shown to reduce interruption burden and device interaction time without adversely affecting the high temporal density of subjective data collection (19–21). The miEMA approach, originally developed by Intille and colleagues (21), involves the delivery of a single question per interruption answered at a glance with a single tap, thereby creating the microinteraction. Questions are cognitively simple with limited response options (e.g., yes/no, scale of 1 to 10). Prompts are delivered through a wearable platform, such as a smartwatch, that is compatible with the individual’s daily life. Questions and response options are structured in a way to require minimal mental effort and must be simple enough to fit on the smartwatch screen without requiring scrolling or compromising readability (13).

The miEMA approach has shown promise in research conducted in civilian populations (13, 19, 21). However, it is unknown if the miEMA experience sampling method is feasible in active duty military sample receiving outpatient TBI rehabilitation, including those undergoing cognitive rehabilitation. Requirements of daily military duties, including both structured routines that differ from civilian occupational routines and interruptions to routines to perform military-related activities (e.g., field training, range operations, training exercises) which may interfere with intensive miEMA data collection. Given the types of military specialties, operational tempo, and general requirements of military service, it is unknown if military personnel will have similar rates of adherence to civilian populations. This pilot study evaluated the feasibility and usability of miEMA delivered through a smartwatch in a sample of military personnel recruited from an outpatient, interdisciplinary TBI program. The sample consisted of two groups completing either a 2-week or 3-week trial of miEMA data collection. The purpose of the 3-week study arm, which consisted of patients concurrently undergoing cognitive rehabilitation, was to also evaluate treatment adherence through individualized Cognitive Survey prompts; however, these results are beyond the scope of the current manuscript.

## 2 Materials and methods

### 2.1 miEMA application overview

**Figure 1:**
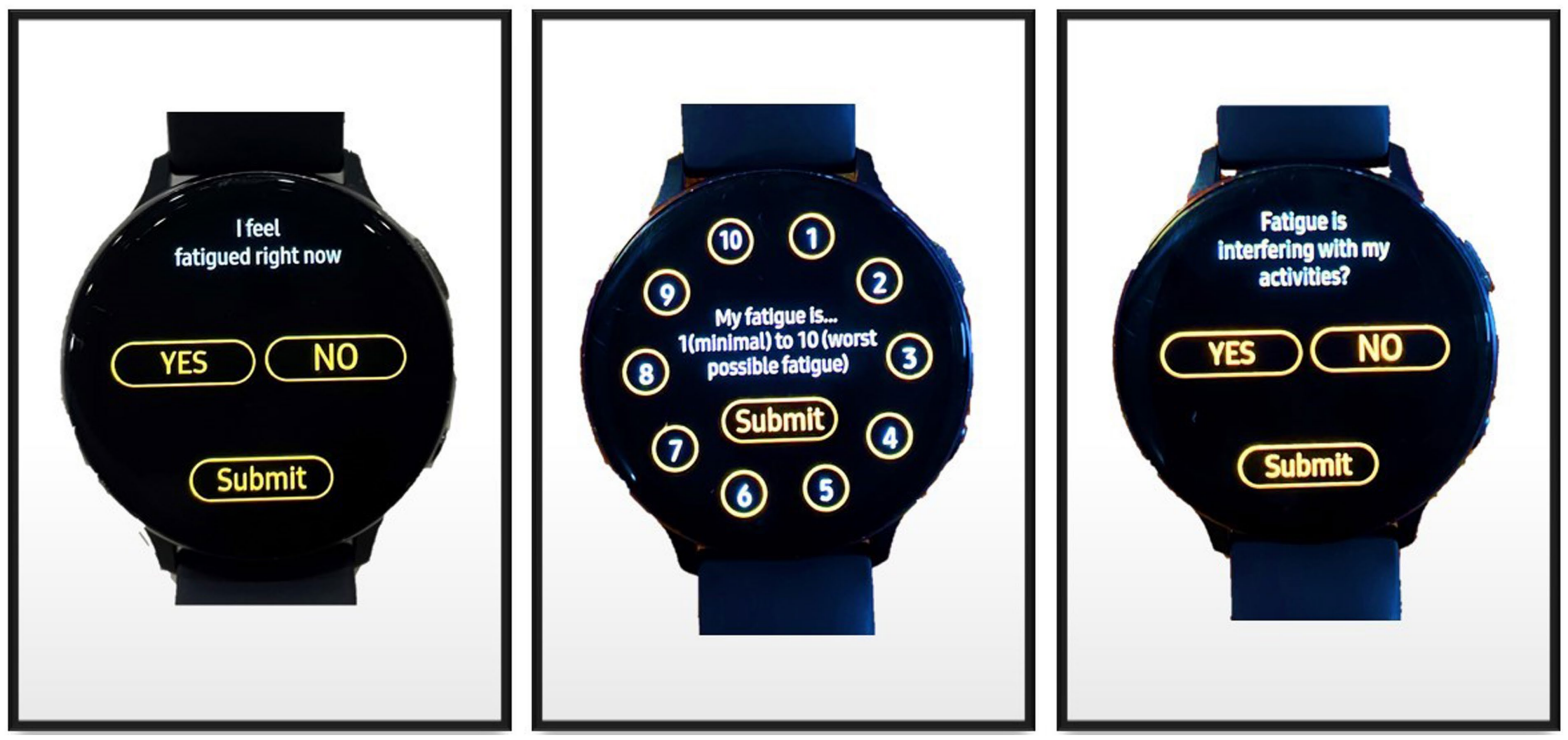
Microinteraction Ecological Momentary Assessment Questions from Fatigue Survey on Smartwatch Face.

The regional MTF’s Clinical Informatics team developed a custom miEMA application (app) called the Clinical Symptom Tracking & Assessment in Real Time (C-STAR) that operates on a smartwatch (Samsung Galaxy Active 2 with a 44 mm watch face) running on the Tizen Operating System. Figure 1 shows example questions displayed on the smartwatch face delivered through the C-STAR app. The decision to develop a custom app was due to the lack of commercial miEMA smartwatch app options available at the time. The C-STAR system has two components: the smartwatch and a secure online survey platform. Select constructs evaluated with miEMA surveys included mood, fatigue, pain, headache, self-efficacy, and cognitive strategy use. All miEMA surveys, except for the individualized Cognitive and Self-efficacy Survey prompts were readily downloaded from the online survey platform to the smartwatch by research staff. Due to the personalized nature of the Cognitive and Self-efficacy Survey prompts, the app engineer had to upload the selected prompts to the C-STAR online survey platform and a research team member then downloaded them to the smartwatch. Surveys were scheduled on the smartwatch when the research staff member selected the surveys for the participant in the “Survey Setup” feature of the app. At the end of each week of the participant’s smartwatch trial, the participant’s response data was converted to a Quick Response code on the C-STAR app for easy and safe transfer from the smartwatch to a Defense Health Agency (DHA) computer using a bar code scanner plugged in via USB port. Survey data were converted to an Excel file (Microsoft 365, Version 2,402) for analysis. The C-STAR app does not require pairing to a mobile device or smartphone and only requires the internet to download new surveys from the online survey platform to the smartwatch. The Bluetooth and Wi-Fi functions were turned off prior to returning the smartwatch to the participant.

All survey questions met miEMA criteria: (1) one question presented at a time, (2) structured to be answered at a glance (within 3 to 5 seconds (s)), and (3) structured to be answered with one tap (yes/no or scale type questions) to create the microinteraction (13). Participants were required to tap a *“Submit”* button that appeared on the smartwatch screen simultaneously with the response options to record their response. An example of a survey question as displayed on the smartwatch is presented in Figure 1. When the prompt appeared on the smartwatch face, the smartwatch alerted the participant that a prompt was delivered (first alert) with a vibrotactile pattern (no audio) of continuous intensity lasting approximately 5 s. The vibrotactile pattern consisted of one 1-s pulse vibration, 1-s pause, four short vibrating pulsations over 1 s, 1-s pause, and then one 1-s pulse vibration. Vibrations paused for 5 s, then if the participant had not yet responded, the smartwatch would vibrate with a pattern of two 1-s pulse vibrations of continuous intensity (second alert). The prompt remained on the screen until the participant submitted a response or until 20 s passed since the first alert. If the participant entered their response, they had to then tap the *“Submit”* button, which appeared on the screen simultaneously with the response options. A response could be changed until the participant tapped the *“Submit”* button. Once the participant tapped the *“Submit”* button, a message confirming their response was submitted appeared on the smartwatch screen stating, *“Response Recorded At (time).”* If a participant did not answer the prompt after the second alert, then the prompt disappeared after 20 s since the first alert and a message appeared informing the participant, “*Previous Survey Was Missed*.” The only functions enabled on the smartwatch while the participant was wearing it were the time, heart rate, and the miEMA app. All other functions were disabled or removed before issuing the smartwatch to the participant.

### 2.2 miEMA prompt scheduling

We selected a time-contingent sampling strategy so that the miEMA survey prompts for each construct were scheduled to be delivered over 10 h of the day (8 a.m. – 6 p.m.) coinciding with the regular duty day and included weekends. The 10-h day was categorized into four 2.5-h blocks of time (henceforth, referred to as “windows”). The windows were categorized as 8 a.m. to 10:30 a.m. (early morning; window 1), 10:30 a.m. to 1 p.m. (late morning/early afternoon; window 2), 1 p.m. to 3:30 p.m. (afternoon; window 3), and 3:30 p.m. to 6 p.m. (late afternoon/evening; window 4). The timing of the delivery of each survey was randomized within each window. Prompts were delivered even if the smartwatch was being charged or not being worn by the participant. Only the initial questions from each survey set and every Cognitive Survey prompt were scheduled. Follow-up questions in survey sets were not scheduled, but their delivery was contingent upon the participant’s response behavior. For example, the initial prompt for the Headache Survey (*“I have a headache”*) was a scheduled prompt; however, delivery of the first follow-up prompt (*“My headache is…”*) was triggered only if the participant responded “yes” to the initial question. The delivery of the second follow-up question in the Headache Survey set (*“Headache is interfering with my activities?”*) was only delivered if the participant submitted a response to the second question.

Participants were instructed not to respond to a prompt if doing so would incur risk (i.e., while driving, performing risky military activities, or operating machinery). If a participant missed an initial question in a survey set, then a question retry mechanism was activated and the missed question was delivered a second time within the same window of time, within about 15 min. The retry mechanism offered the participant a second opportunity to respond to a missed initial question in a survey set. Retry questions and follow-up questions were not scheduled but were delivered based on the participant’s response behavior. If the participant missed the retry question, the question was not delivered a third time, but was recorded as another missed response. Follow-up questions in survey sets did not have a built-in retry mechanism; therefore, if a participant did not answer a follow-up question, the prompt was recorded as having a missed response and any additional follow-up questions in the survey set, if applicable, were not delivered. Cognitive Survey prompts also did not have a built-in retry mechanism; therefore, if a participant did not answer a Cognitive Survey prompt, the prompt was recorded as a missed response. Cognitive Survey prompts were scheduled as two separate surveys since the prompts addressed two separate skills or behaviors; therefore, the delivery of these prompts was not contingent upon a response to a previous Cognitive Survey prompt. For example, if a participant missed the first Cognitive Survey prompt, then it was recorded as a missed response, but the second Cognitive Survey prompt was still delivered.

Each of the selected survey sets was scheduled to be delivered once during each of the four windows, except for the Self-efficacy and Alcohol Surveys which were only scheduled to be delivered once a day during the early morning window (window 1). The timing of survey delivery during each window was randomized to minimize non-response bias and improve the generalizability of the data collected over the trial in various contexts. For example, if a participant was administered the Mood Survey (two questions), Headache Survey (three questions), and Cognitive Survey (two prompts), then each survey would be initiated once during each window, or four times daily, but the timing of the delivery of each survey was randomized during each window. The number of prompts delivered was based on which miEMA surveys were selected, how many questions were in the selected survey sets, and the participant’s response behavior. In the example described, if a participant responded “yes” to every initial Headache Survey question, thus endorsing headache every time, responded to the two follow-up Headache Survey questions, and responded to every question delivered for both the Mood and Cognitive Surveys, then the total number of delivered questions would be seven for each window, or 28 questions in 1 day, and 196 questions over 7 days.

### 2.3 Study design and participants

#### 2.3.1 Study design

This was a prospective study with temporally-dense repeated measures delivered through a custom miEMA smartwatch app over two or three weeks.

#### 2.3.2 Participants

Participants were 28 United States (U.S.) service members enrolled at a military outpatient interdisciplinary TBI rehabilitation center at the Madigan Army Medical Center, Joint Base Lewis-McChord, Washington. Participants in the 3-week study arm were patients at the TBI center who were referred by their cognitive rehabilitation provider. Participants were included in the study if they were (1) currently active duty military, Reserves, or National Guard; (2) between the ages of 20 and 65 years; (3) self-reported a history of TBI that occurred during military service; (4) reported any of the following persistent TBI-related symptoms: headache, mood changes, fatigue or energy loss, sleep difficulties, and/or cognitive difficulties; and (5) were able and willing to wear a smartwatch throughout the day including while on duty (for 14 days for the 2-week arm and for 21 days for the 3-week arm). Additional inclusion criteria for the 3-week participants were: (1) currently enrolled in cognitive rehabilitation treatment at the clinic and demonstrated commitment and (2) interest in changing or learning more about their own performance, as determined by their cognitive rehabilitation provider. Individuals who (1) endorsed active suicidal ideation or psychotic symptoms or (2) were concurrently enrolled in a specialty behavioral health intensive outpatient program or specialty substance abuse program were excluded from participating. All participants had at least one clinically diagnosed TBI verified in their medical record. The protocol for this study was reviewed and approved by the Madigan Army Medical Center Institutional Review Board (Protocol # 222049).

### 2.4 Study procedures

Once research staff determined an individual’s eligibility to participate in the study, participants were enrolled in one of two study arms. Participants enrolled in the 3-week study arm were patients undergoing interdisciplinary TBI rehabilitation and referred by their cognitive rehabilitation provider due to current engagement in cognitive rehabilitation for TBI. Participants enrolled in the 2-week study arm were also patients undergoing interdisciplinary TBI rehabilitation but were not concurrently undergoing cognitive rehabilitation for TBI. Upon completing consenting procedures, research staff collected demographic information, administered the Neurobehavioral Symptom Inventory (NSI), and elicited lifetime TBI history through a semi-structured interview using the Ohio State University TBI-Identification Method. All participants attended a 15-min Smartwatch Orientation in which the research staff member briefed the participant on the functionality of the smartwatch, including how to operate and navigate the smartwatch interface. Each participant was shown and then asked to demonstrate their ability to respond to questions on the miEMA app. Every participant was given a “Smartwatch and miEMA Orientation” handout for reference before departing the orientation. Contact information was provided to each participant should they encounter any technical issues with the smartwatch at any time during the trial. Upon conclusion of the orientation, the participant received the smartwatch and charger to take home for the duration of their study participation. Participants were instructed to wear the smartwatch from 8 a.m. to 6 p.m. daily on their preferred wrist.

All participants were required to attend weekly research appointments at the TBI center which were scheduled during the Smartwatch Orientation. Weekly research appointments for the 3-week trial participants coincided with their clinical visits with their cognitive rehabilitation provider so that they returned the smartwatch to research staff before their clinical visit and retrieved the smartwatch after their clinical visit. Weekly research appointments for the 2-week trial participants coincided with their clinical visits with providers at the TBI center so that they returned the smartwatch to research staff before their clinical visit and retrieved the smartwatch after their clinical visit. During their clinical visit, research staff downloaded response data from the smartwatch and uploaded a new schedule of prompts. Participants were instructed to charge the smartwatch any time between 6 p.m. and 8 a.m., which typically coincided with sleep hours, so as not to conflict with smartwatch wear during the hours of miEMA prompt delivery. Participants were compensated for their time with a $25 gift card upon concluding their study participation and returning the smartwatch and charger. Participants were not directly compensated for high adherence or for meeting any prescribed adherence goals.

### 2.5 miEMA, self-report, and usability measures

Detailed descriptions of the following measures are presented in the Supplementary materials.

#### 2.5.1 miEMA surveys

Characteristics of each miEMA survey are presented in Table 1. The miEMA survey set options in the C-STAR app included Mood (two prompts), Alcohol (one prompt), Self-efficacy (one prompt), Headache (three prompts), Fatigue (three prompts), Pain (three prompts), and Cognitive (two prompts delivered as two separate survey sets) Surveys. The Mood, Alcohol, Headache, Fatigue, and Pain Surveys were standardized survey sets. The Cognitive Surveys assessed cognitive strategy use and were individualized to the participant. The provider and participant co-selected the Cognitive Survey statements from a 120-item bank based on the participant’s specific challenges and treatment goals. The Self-efficacy Survey statements were created by the research staff member based on the aspect of Self-efficacy the participant wanted to monitor (i.e., *“I can engage in daily activities*”).

**Table 1:** Microinteraction ecological momentary assessment (miEMA) survey structure.

Survey	Prompt description	Response options	Scheduled?	Delivery response-contingent?
Alcohol	*1. I drank alcohol yesterday.*	Yes/No	Yes	No
	2. *How many standard drinks did you have yesterday?*	1–2, 3–4, 5–6, >6	No	Yes
Self-efficacy	1. Personalized, symptom-based statement	1 to 10 (not confident to very confident)	Yes	No
Mood	1. *My mood is?*	1 to 8 (negative to positive)	Yes	No
	2. *My arousal level is?*	1 to 8 (low to high)	No	Yes
Headache	1. *I have a headache.*	Yes/No	Yes	No
	2. *My headache is...*	1 to 10 (very mild to very severe)	No	Yes
	3. *Headache is interfering with my activities?*	Yes/No	No	Yes
Fatigue	1. *I feel fatigued right now.*	Yes/No	Yes	No
	2. *My fatigue is...*	1 to 10 (very mild to very severe)	No	Yes
	3. *Fatigue is interfering with my activities?*	Yes/No	No	Yes
Pain	1. *I am in pain right now.*	Yes/No	Yes	No
	2. *My pain is...*	1 to 10 (very mild to very severe)	No	Yes
	3. *Pain is interfering with my activities?*	Yes/No	No	Yes
Cognitive	1. Treatment-related statement	Yes/No	Yes	No
	2. Treatment-related statement	Yes/No	Yes	No

Self-efficacy and Cognition Surveys were individualized to the participant’s symptoms and treatment goals. The clinician and/or study staff determined which miEMA surveys were given to the participant based on their reported symptoms and concerns. The participant decided which aspect of self-efficacy they wanted to monitor for the Self-efficacy Survey. The Cognition Survey statements were chosen collaboratively by the Speech-Language Pathologist and the participant from a 120-item bank based on treatment goals.

All participants were given the miEMA Mood Survey. Research staff used the participant’s NSI responses as well as participant input to determine which additional miEMA surveys would be most relevant. Participants in the 3-week study arm completed individualized Cognitive Surveys to monitor treatment adherence in conjunction with cognitive rehabilitation.

#### 2.5.2 Neurobehavioral symptom inventory

The NSI is a 22-item measure of post concussive symptoms, including headache, fatigue, pain, and mood. Individuals rate the severity of their symptoms over the past month. Responses range from 0 to 4 (0, none; 1, mild; 2, moderate; 3, severe; and 4, very severe), with higher scores indicating more severe symptoms (22). Internal consistency of the NSI is high with a coefficient alpha range from 0.88 to 0.92 (23).

#### 2.5.3 Mobile health application usability questionnaire (MAUQ)

The MAUQ assesses the usability of mobile health apps (24, 25). The full original measure contains 21 items categorized into three subscales. The current study used 12 items that break out into two subscales: (1) Ease of Use and Satisfaction (EOU) and (2) System Information Arrangement (SIA). Each item is rated on a Likert scale from 1 (strongly disagree) to 7 (strongly agree). The language for the MAUQ items was slightly modified for the current study to align with app use and is presented in Supplementary Table S1. The overall MAUQ has strong internal consistency with a Cronbach alpha = 0.932, as well as does the EOU, Cronbach alpha = 0.85, and SIA, Cronbach alpha = 0.91 (25).

#### 2.5.4 System usability scale (SUS)

The SUS is a 10-item measure used to investigate human factors associated with interfacing with new information technology (26). Individuals rate their level of agreement with statements ranging from 1 (strongly disagree) to 5 (strongly agree). The SUS items can be categorized into two subscales: (1) Usable and (2) Learnable (27). Standard score conversion procedures were used to convert raw scores ranging from 0 to 40 to scores that range from 0 to 100 (28). Internal consistency for the SUS has ranged from 0.70 to 0.95 (29).

### 2.6 Response behavior measures

Participant adherence was measured as completion and compliance rates as previously defined by Ponnada and colleagues (13). We further characterized response behavior based on the use of the retry mechanism, survey set completion, survey set completion based on time of day, and Cognitive Survey set completion (3-week study arm only).

#### 2.6.1 Question-response characteristics

The prompting frequency (number of prompts delivered per window), number of daily interruptions, questions delivered, responses, possible questions in a survey set, scheduled questions, responses to scheduled questions, and retry questions delivered were measured.

#### 2.6.2 Completion

Completion was defined as the percentage of questions answered out of questions delivered. This definition of completion is commensurate with Ponnada and colleagues’ (13) definition of completion from their previous research.


$$\begin{array}{l} \mathrm{Completion rate}\;\left(\%\right)=\#\mathrm{Questions answered}/\#\mathrm{Questions}\;\\ \mathrm{delivered}\;x\;100 \end{array}$$

#### 2.6.3 Compliance

Compliance was defined as the percentage of questions answered out of questions scheduled which was also based on Ponnada and colleagues’ (13) previous definition. For our miEMA app, only the initial questions from each survey set and all Cognitive Survey prompts were scheduled. Retry and follow-up questions were delivered according to participant response behavior and were not scheduled, therefore, these questions were not included in the denominator for compliance.


$$\begin{array}{l} \mathrm{Compliance rate}\;\left(\%\right)=\#\mathrm{Questions answered}/\#\mathrm{Questions}\;\\ \mathrm{scheduled}\;x\;100 \end{array}$$

#### 2.6.4 Mean participant completion

The mean participant completion rate is an average of all participant completion rates across the sample and for each study arm.

#### 2.6.5 Mean participant compliance

The mean participant compliance rate is an average of all participant compliance rates across the sample and for each study arm.

#### 2.6.6 Mean participant completion using retry mechanism

Mean participant completion using the built-in retry mechanism was defined as the mean percentage of questions answered out of questions delivered, excluding initial questions that were not answered and including retry questions defined as questions that were delivered a second time in the same time window via the retry mechanism. For example, if the participant did not respond to the initial prompt in the Headache Survey (*“I have a headache”*), then the same question was delivered a second time during the same window using the retry mechanism. The initial unanswered prompt would not count in the denominator, but the retry question delivered using the retry mechanism would count in the denominator whether it was answered or not.


$$\begin{array}{l} \begin{array}{l} \mathrm{Completion rate using}\\ \;\mathrm{retry mechanism}\;\left(\%\right)=\#\mathrm{Questions answered}/(\#\mathrm{Questions}\;\\ \mathrm{delivered}-\#\mathrm{Initial} \end{array}\\ \begin{array}{l} \mathrm{questions not answered then}\;\\ \mathrm{delivered again}\;via\;\\ \mathrm{retry mechanism})\;x\;100 \end{array} \end{array}$$

#### 2.6.7 Mean participant survey set completion

To calculate survey set completion, we first accounted for the total number of possible questions in a survey set based on the number of questions in each survey set for each participant and the duration of their smartwatch trial. Mean participant survey set completion was defined as the mean percentage of questions answered out of total possible questions in a complete survey set, including those not delivered because the preceding question in the survey set was not answered, but excluding initial unanswered questions that were delivered again via the retry mechanism. We also excluded follow-up questions in a survey set that were not delivered because the participant responded “no” to the initial question (i.e., *“I feel fatigued right now”*) since the follow-up questions would not have been delivered (i.e., *“My fatigue is…”* and *“Fatigue interfering with my activities?*”).


$$\begin{array}{l} \mathrm{Survey}\;\mathrm{set}\;\\ \mathrm{completion rate}\;\left(\%\right)=\#\mathrm{Questions answered}/\#\mathrm{Possible}\;\\ \mathrm{questions in}\;a\;\mathrm{survey}\;\mathrm{set}\;x\;100 \end{array}$$

#### 2.6.8 Mean participant survey set completion based on time of day

We also stratified survey set completion based on the time of day the survey was delivered. We defined survey set completion as described above, then stratified completion rates based on the time of day (Windows 1 through 4).


$$\begin{array}{l} \begin{array}{l} \mathrm{Survey}\;\mathrm{set}\;\mathrm{completion}\;\\ \mathrm{rate based}\;\mathrm{on}\;\mathrm{time of}\;\mathrm{day}\;\left(\%\right)=\#\mathrm{Questions answered}/\#\mathrm{Possible}\;\\ \mathrm{questions in}\;a \end{array}\\ \begin{array}{l} \mathrm{survey}\;\mathrm{set}\;\mathrm{delivered in}\;a\;\\ \mathrm{specified time window}\;x\;100 \end{array} \end{array}$$

#### 2.6.9 Mean participant cognitive completion

For the 3-week study arm participants, mean participant cognitive completion was calculated as the mean percentage of responses to the Cognitive Survey prompts out of the number of Cognitive Survey prompts delivered. Participants in the 3-week study arm and their provider selected two cognitive statements per week based on their individual cognitive challenges and goals. All cognitive prompts were scheduled as individual surveys; therefore, the delivery of one cognitive prompt was not contingent upon a response to another cognitive prompt.


$$\begin{array}{l} \mathrm{Cognitive}\;\\ \mathrm{completion rate}\;\left(\%\right)=\#\mathrm{Cognitive responses}/\#\mathrm{Cognitive}\;\\ \mathrm{prompts delivered}\;x\;100 \end{array}$$

## 3 Results

### 3.1 Demographics and miEMA surveys

#### 3.1.1 Demographics

Descriptive statistics for select demographics and the types of miEMA Surveys given to participants in each study arm are presented in Table 2. Given the very small group sizes, the similarity in demographics between study arm groups, and the purpose of evaluating treatment adherence in the 3-week study arm that is beyond the scope of this manuscript, we did not perform statistical comparisons between the 2-week and 3-week groups. Of 30 participants enrolled in the study, 28 (93%) completed the smartwatch trial with 15 participants in the 2-week arm and 13 participants in the 3-week arm. Two participants from the 3-week arm withdrew from the study after enrollment. One participant withdrew before starting the smartwatch trial due to a military training conflict and another withdrew after completing 1 week of the smartwatch trial due to a family emergency. The data collected from the withdrawn participant during that 1 week was not included in the analysis. The overall sample had a mean age of 34.9 ± 8.5 years and 14.2 ± 2.3 years of education. Participants were mostly white (68%), male (86%), serving in the Army (89%), and primarily enlisted (89%) who were serving in a combat support military occupational specialty (MOS, 71%).

**Table 2:** Demographic and survey administration characteristics of participants by study arm.

Characteristic	Overall (*N* = 28)		2-week arm (*n* = 15)		3-week arm (*n* = 13)	
	Mean	SD	Mean	SD	Mean	SD
Age (years)	34.9	8.5	35.3	9.7	34.5	7.2
Education (years)	14.2	2.3	13.9	2.2	14.6	2.4
	N	%	n	%	n	%
Race						
White	19	68	9	60	10	77
Black/African American	3	11	1	7	2	15
Asian	1	4	1	7	0	0
Native Hawaiian/Pacific Islander	1	4	1	7	0	0
Hispanic/Latino	4	14	3	20	1	8
Sex						
Female	4	14	2	13	2	15
Male	24	86	13	87	11	85
Branch of service						
Army	25	89	14	93	11	85
Air Force	1	4	0	0	1	8
Navy	2	7	1	7	1	8
Rank						
Enlisted	25	89	14	93	11	85
Officer	3	11	1	7	2	15
MOS						
Support MOS	20	71	13	87	7	54
Combat MOS	8	29	2	13	6	46
miEMA surveys administered to participants						
Self-efficacy survey	6	21	4	27	2	15
Headache survey	17	61	11	73	6	46
Mood survey	28	100	15	100	13	100
Fatigue survey	12	43	7	47	5	39
Pain survey	6	21	5	33	1	8
Cognitive survey	14	50	1	7	13	100

SD, standard deviation; MOS, Military Occupational Specialty; miEMA, Microinteraction Ecological Momentary Assessment.

#### 3.1.2 miEMA surveys

Most participants (*n* = 25, 89%) were administered three miEMA surveys. Two participants from the 2-week study arm were given only two surveys each and one participant from the 3-week study arm selected four surveys. All participants were administered the Mood Survey. All 3-week arm participants were given a Cognitive Survey tailored to their cognitive rehabilitation treatment plan and goals. One 2-week arm participant selected the individualized Cognitive Survey based on their personal treatment goals. Of the remaining miEMA surveys, 61% (*n* = 17) of participants selected the Headache Survey across the sample. None of the participants opted to complete the Alcohol Survey.

### 3.2 Response behavior

Characteristics of questions and response metrics are presented in Table 3. Participants received a mean of 23.4 ± 5.4 (range: 12–36) prompts per day depending on how many and which surveys they were given. The average number of prompts delivered to participants during each window was 5.9 ± 1.4 (range: 3–9) and were also dependent upon how many and which surveys participants were given. Descriptive statistics for each type of completion and compliance for the overall sample and for each study arm stratified by week are presented in Table 4.

**Table 3:** Characteristics of questions and response metrics for the sample across all weeks and by study arm.

	Overall (*N* = 28)		2-week arm (*n* = 15)		3-week arm (*n* = 13)	
	Mean	SD	Mean	SD	Mean	SD
Number of interruptions per day	23.3	5.2	22.9	6.2	23.8	3.9
Prompting frequency (number of questions per 2.5-hour window)	5.8	1.3	5.7	1.5	6.0	1.0
	No.		No.		No.	
Delivered questions^a^	11,320		4,810		6,510	
Responses	9,071		3,935		5,136	
Scheduled questions^b^	6,395		2,284		4,111	
Responses to scheduled questions^b^	4,952		1,809		3,143	
Possible questions^c^	11,335		4,885		6,450	
Cognitive survey prompts delivered	2,220		104		2,116	
Cognitive survey responses	1,723		91		1,632	
	No.	%	No.	%	No.	%
Retry questions delivered	905	8.0	444	9.2	461	7.1
	Mean	SD	Mean	SD	Mean	SD
Retry questions delivered per participant	32	25	30	28	35	21

^aIncludes initial questions that were unanswered and retry questions that were delivered. bOnly initial questions in a survey set were scheduled. Follow-up questions in survey sets were not scheduled. cThe number of possible questions in a survey set, including follow-up questions that were not delivered due to the participant not responding to a previous question in the survey set. Initial questions that were not answered but then delivered again in the same time window using the retry mechanism were not included. Follow-up questions that were not delivered due to a preceding “no” response to the initial survey question were not included. No., number; SD, standard deviation.^

**Table 4:** Completion and compliance across the sample by week and by study arm.

Weeks	Overall (*N* = 28)		2-week arm (*n* = 15)		3-week arm (*n* = 13)	
Completion^a^	%		%		%	
Week 1	82.2		84.4		79.9	
Week 2	79.7		79.5		80.0	
Week 3	–		–		76.7	
Weeks combined	80.1		81.8		78.9	
Compliance^b^	%		%		%	
Week 1	79.4		81.1		78.1	
Week 2	77.7		77.6		77.8	
Week 3	–		–		73.4	
Weeks combined	77.4		79.2		76.5	
Mean participant completion	%	SD (%)	%	SD (%)	%	SD (%)
Week 1	82.2	12.3	84.9	13.3	79.1	10.7
Week 2	81.0	14.2	81.2	15.5	80.7	13.1
Week 3	–	–	–	–	76.3	14.6
Weeks combined	81.1	12.0	83.0	13.8	78.9	9.5
Mean participant compliance						
Week 1	79.6	12.6	81.0	15.0	78.1	9.5
Week 2	78.4	15.8	78.2	17.5	78.6	14.2
Week 3	–	–	–	–	73.1	15.0
Weeks Combined	78.1	13.0	79.5	15.7	76.6	9.3
Mean participant completion using retry mechanism^c^						
Week 1	88.4	9.2	91.6	8.5	84.6	8.9
Week 2	87.6	10.5	89.0	11.2	86.1	9.8
Week 3	–	–	–	–	82.1	12.1
Weeks combined	87.7	8.8	90.3	9.3	84.6	7.5
Mean participant survey set completion^d^						
Week 1	82.7	13.7	84.8	15.2	80.4	11.9
Week 2	82.0	15.8	81.3	18.1	82.7	13.4
Week 3	–	–	–	–	76.1	14.1
Weeks combined	81.5	13.3	82.9	16.2	79.8	9.4
Mean participant cognitive completion^e^						
Week 1	–	–	–	–	77.8	11.4
Week 2	–	–	–	–	77.7	15.3
Week 3	–	–	–	–	75.8	15.6
Weeks combined	–	–	–	–	77.1	11.6

^aPercentage of questions answered out of questions delivered. bPercentage of questions answered out of questions scheduled. Only initial questions in survey sets and all Cognitive Survey prompts were scheduled. cMean percentage of questions answered out of questions delivered, excluding initial questions that were not answered and then delivered a second time in the same window via the retry mechanism. dMean percentage of questions answered out of number of questions in a complete survey set, including follow-up questions not delivered because the preceding question in the survey set was not answered, but excluding follow-up questions not delivered due to a “no” response to an initial question. eMean percentage of responses to cognitive prompts out of cognitive prompts delivered. Includes only 3-week study arm participants who were concurrently undergoing cognitive rehabilitation. SD, standard deviation.^

#### 3.2.1 Completion

A total of 11,320 prompts were delivered through the C-STAR app and 9,071 responses were collected across the sample. The overall completion rate was 80.1% across the sample. Completion marginally decreased from the first to the second week across the entire sample. In the 3-week study arm, completion reduced by 3.3% from the second to the third weeks. Overall completion was slightly higher in the 2-week study arm than the 3-week study arm.

#### 3.2.2 Compliance

A total of 6,395 questions were scheduled to be delivered through the C-STAR app with a total of 4,952 responses to scheduled questions for a compliance of 77.4% across the sample. Compliance decreased a small percentage from the first to the second week in both study arms. Compliance further decreased from Week 2 to Week 3 in the 3-week study arm by 4.4%. Compliance was marginally higher in the 2-week study arm than the 3-week study arm.

#### 3.2.3 Mean participant completion

Mean participant completion was higher in the 2-week study arm than the 3-week study arm for the weeks combined. The mean participant completion rate was 81.1% ± 12.0% across the sample, with a mean difference of 4.1% between study arms. There was a general decline in completion from Week 1 to Week 2 in the 2-week group, in contrast to the slight increase in the 3-week group. However, mean participant completion in the 3-week group declined minimally between Weeks 2 and 3.

#### 3.2.4 Mean participant compliance

Mean participant compliance was slightly higher in the 2-week study arm than the 3-week study arm for the weeks combined. The mean participant compliance rate was 82.2% ± 12.3% across the sample, with a 5.8% mean difference between study arms. Compliance decreased from the first to the second week in the 2-week study arm and slightly increased in the 3-week study arm.

#### 3.2.5 Mean participant completion using the retry mechanism

A total of 905 initial questions in survey sets were not answered upon first-time delivery, therefore, these prompts were delivered a second time in the same window via the retry mechanism. Retry questions comprised 8.0% of all delivered questions for the sample across all weeks combined (median = 25 retry questions per participant). Mean participant completion using the retry mechanism was 87.7% ± 8.8% across the sample for all weeks combined. The 2-week study arm had a higher mean completion using the retry mechanism (90.3% ± 9.3%) than the 3-week study arm (84.6% ± 7.5%). The implementation of the retry mechanism increased mean completion rates by 6.6% overall across the entire sample.

#### 3.2.6 Mean participant survey set completion

We accounted for a total of 11,335 possible questions based on number of questions in each survey set, the response behavior of each participant, and the duration of each participant’s smartwatch trial. Mean participant survey set completion was 81.5% ± 13.3% across the sample for all weeks combined. Survey set completion slightly decreased in the 2-week study arm and increased in the 3-week study arm. There was a decrease in survey set completion in the 3-week study arm from Week 2 to Week 3. The 2-week study arm mean participant survey set completion rate was higher than the 3-week study arm across weeks combined.

#### 3.2.7 Mean participant survey set completion based on time of day

Survey set completion for each window across the sample and by study arm is presented separately in Table 5. Mean participant survey set completion rates during each window across the sample for all weeks combined were as follows: Window 1 (early morning) 69.8 ± 18.3%; Window 2 (late morning/early afternoon) 85.7 ± 12.8%; Window 3 (afternoon) 86.2 ± 12.6%; and Window 4 (late afternoon/evening) 85.0 ± 14.7%.

**Table 5:** Mean participant survey set completion based on window (Time of Day) across the sample and by study arm.

Window, Time of Day	Overall (*N* = 28)		2-week arm (*n* = 15)		3-week arm (*n* = 13)	
	%	SD (%)	%	SD (%)	%	SD (%)
Window 1 Early morning, 8:00 a.m. to 10:30 a.m.	69.8	18.3	71.2	19.5	68.1	17.4
Window 2 Late morning/early afternoon, 10:30 a.m. to 1:00 p.m.	85.7	12.8	86.4	16.0	85.0	8.3
Window 3 Afternoon, 1:00 p.m. to 3:30 p.m.	86.2	12.6	88.5	15.7	83.5	7.6
Window 4 Late afternoon/early evening, 3:30 p.m. to 6:00 p.m.	85.0	14.7	86.4	18.9	83.3	8.0

Survey set completion is the percentage of questions answered out of possible questions in a survey set delivered in the specified time window.

#### 3.2.8 Mean participant cognitive completion

A total of 2,116 Cognitive Survey prompts were delivered to participants in the 3-week study arm with a total of 1,632 responses. Mean participant completion of Cognitive Survey prompts was 77.1% ± 11.6%. This completion rate was consistent across each of the 3 weeks (Week 1: 77.8% ± 11.4%; Week 2: 77.7% ± 15.3%; Week 3: 75.8% ± 15.6%). A single participant in the 2-week study arm requested to do a Cognitive Survey throughout their smartwatch trial; however, since this participant was not concurrently undergoing cognitive rehabilitation, these data were not included in the mean participant cognitive completion analysis.

### 3.3 Usability

Results of the MAUQ and SUS are presented in Table 6.

**Table 6:** Usability survey scores and percentile rankings across sample and by study arm.

Questionnaire	Overall (*N* = 28)		2-week arm (*n* = 15)		3-week arm (*n* = 13)	
Mobile health application usability questionnaire (MAUQ)	Mean	SD	Mean	SD	Mean	SD
Overall item score	6.3	1.1	6.3	1.2	6.2	1.2
Subscale item score – ease of use and satisfaction	6.5	0.9	6.6	0.8	6.4	0.9
Subscale item score – system information arrangement	5.9	1.5	5.9	1.5	5.9	1.5
System usability scale (SUS)	Mean	SD	Mean	SD	Mean	SD
Overall score	89.0	7.2	86.5	6.8	92.1	6.7
Percentile rankings (%)	96.4	8.4	94.5	10.9	98.7	2.2
Usable subscale	87.3	8.3	84.9	6.9	90.0	8.4
Learnable subscale	95.8	9.8	92.5	12.3	100.0	0

Item scores for the Mobile Health Application Usability Scale (MAUQ) range from 1 (strongly disagree) to 7 (strongly agree), with higher item scores indicating higher perceived usability. For the System Usability Scale (SUS), the standard score conversion procedure was used to convert each participant’s responses to a score between 0 and 100 for the overall SUS and for each subscale. A higher SUS score indicates higher perceived usability. The overall converted score was used to determine a percentile ranking. The percentile ranking indicates the percentage of applications in the SUS database that the current application had a higher perceived usability. SD, standard deviation.

#### 3.3.1 MAUQ results

The mean overall item score for the MAUQ was 6.3 ± 1.1 across the sample, indicating excellent overall usability of the C-STAR app. Overall MAUQ mean item scores for the 2-week and 3-week study arms were 6.3 ± 1.2 and 6.2 ± 1.2, respectively. Mean item scores for the MAUQ EOU subscale were 6.5 ± 0.9 for the sample, 6.6 ± 0.8 for the 2-week study arm, and 6.4 ± 0.9 for the 3-week study arm, indicating excellent ease of use and satisfaction with the C-STAR app. Mean item score for the MAUQ SIA subscale was 5.9 ± 1.5 for the sample and both study arms, suggesting acceptability in navigating and interacting with the app.

#### 3.3.2 SUS results

For the SUS, the sample’s mean converted score was 89.0 ± 7.2 out of 100 with a percentile ranking of 96.4 ± 8.4%, indicating excellent perceived system usability. Converted mean SUS scores for the 2-week and 3-week study arms were 86.5 ± 6.8 and 92.1 ± 6.7, respectively. Percentile rankings for the 2-week and 3-week study arms were 94.5% ± 10.9% and 98.7% ± 2.2%, respectively. The mean SUS Usable subscale score was 87.3 ± 8.3 for the sample, 84.9 ± 6.9 for the 2-week study arm, and 90.0 ± 8.4 for the 3-week study arm, indicating high levels of perceived usability of the app and smartwatch system. The mean SUS Learnable subscale score was 95.8 ± 9.8 for the sample, 92.5 ± 12.3 for the 2-week study arm, and 100.0 ± 0 for the 3-week study arm, suggesting that operating the technology was very easy to learn.

## 4 Discussion

The findings from this study complement those published from previous research studies (13, 21) in that we assessed the adherence to and usability of miEMA using a smartwatch for tracking both symptoms and use of cognitive behavioral strategies in service members with a history of TBI. The comparable completion and compliance rates suggest that miEMA was a feasible method of experience sampling in a military TBI population. The temporally dense *in-situ* data was collected over a period of two and three weeks with high adherence. Smartwatches have demonstrated acceptability (30–32) and higher compliance, lower perceived user burden, and faster response times than standard EMA to deliver miEMA questions (19, 21). The current study findings suggest that miEMA surveys delivered through a digital app on a smartwatch is feasible, easy to learn, and practical to use within the unique ecological contexts of the daily military lifestyle of service members in the garrison environment.

### 4.1 Response behavior

#### 4.1.1 Completion and compliance

Previous work has demonstrated completion and compliance rates comparable to the current study’s findings (13, 21). Intille et al. (21) observed 87.8% completion and 75.6% compliance rates in a 4-week pilot study in which 19 participants were randomized to use miEMA delivered through a smartwatch (vs. 14 participants randomized to a standard EMA condition via smartwatch). When two outliers were removed from the Intille et al. (21) analysis, completion increased to 91.8% and compliance to 88.2%. Ponnada et al. (13) conducted another pilot study with 15 participants responding to miEMA questions delivered through a smartwatch over 1 month and observed a 76.4% ± 22.3% completion rate. Ponnada and colleagues’ (13) longitudinal follow-on study assessed miEMA adherence in 81 young adults over at least 6 months of smartwatch data collection and observed a mean participant compliance rate of 67.4% ± 13.7% and completion rate of 80.2% ± 13.3%. The current study also demonstrated minimal differences in completion and compliance between weeks in both study arms suggesting that the longitudinal duration of 14 to 21 days had limited impact on adherence measures. Despite a rate of up to 36 interruptions per day (M = 23.4), our study’s findings suggest that miEMA *in-situ* data collection was manageable and sustainable for the participants for up to 21 days.

It should be highlighted that completion and compliance as previously defined did not fully account for undelivered questions based on nonresponse nor unscheduled follow-up questions in our study. These omissions were likely due to differences in the design of the custom miEMA app in the current study compared to that in previous studies (13, 21). For example, if an initial question (i.e., *“I have a headache”*) was not answered and the retry question was also not answered, then the follow-up questions in the survey set (i.e., *“My headache is…”* and *“Headache is interfering with my daily activities?”*) were not delivered. It was not possible to know in that moment if the participant had a headache or not, and therefore, either one data point was missing (i.e., they did not have a headache) or three data points were missing (i.e., they had a headache, it was a 7 out of 10 in severity, and it was or was not interfering with their daily activities). Regarding compliance, only Cognitive Survey prompts and initial questions in survey sets were scheduled, so retry questions and follow-up questions that were or were not delivered based on response behavior were not factored in when calculating compliance. This definition of compliance did not capture the prompts in which their delivery was contingent upon response behavior (e.g., responses to or missed follow-up questions in survey sets). For example, if a participant responded “yes” to an initial question (i.e., *“I have a headache”*), then two follow-up questions were expected to be delivered, but since these follow-up questions are not scheduled, then they would not be accounted for in the compliance definition whether they were answered or not.

#### 4.1.2 Completion using the retry mechanism

Given the inherent risks of some daily activities and military duties, the retry mechanism allowed the participant a second opportunity during the same allotted time window to respond to a missed initial question in a survey set. The retry mechanism increased completion across the sample by 6.6% suggesting it’s value in increasing completion without compromising the safety of military personnel as they navigate their daily activities and military duties. We opted not to use the retry mechanism for follow-up questions in survey sets to keep the prompt burden at a manageable level. Retry questions comprised 8% of all delivered questions and appeared to boost rather than hinder completion in the current study. This finding is aligned with previous research that showed increased prompting of cognitively simple structured questions through a smartwatch allowed for predictable response time and minimized interruption cost (21).

#### 4.1.3 Survey set completion

Due to the limitations of the definitions for completion and compliance related to our custom app design as discussed above, we developed the definition for survey set completion. Survey set completion accounted for responses to follow-up questions in survey sets, as well as missed responses for undelivered questions based on response behavior. Mean participant survey set completion rates were similar to mean participant completion rates in the current study. However, survey set completion provides a more complete picture of the data that were and were not provided by participants and may be more relevant to the clinical application of a miEMA approach based on our app design.

Mean participant survey set completion based on the time of day was worse during the early morning window (8 a.m. to 10:30 a.m.) and higher for the remainder of the day across the sample. These findings are similar to Ponnada and colleagues’ (33) study in which they observed lower completion rates during the morning window (8 a.m. – 12 p.m., 75% ± 18%) and higher completion rates later in the day (80% ± 14%, 12 p.m. - 4 p.m.; and 81% ± 13%, 4 p.m. - 8 p.m.). EMA methods depend upon the careful timing of assessments (6). Our sampling strategy was based on scheduling prompts to solicit experience data during randomized times across four windows throughout a 10-h day, as this strategy aimed to reduce non-response bias and improve the generalizability of the data collected over time. However, the activities of daily routines may have had an impact on response behavior in our sample.

Military personnel have specific daily routines, which are different from most civilian occupations, as well as intermittent activities that diverge from their daily routines (e.g., range operations, parachute training, military unit functions). Military personnel typically conduct Physical Training in the morning but may also conduct Physical Training midday or in the afternoon, depending on their work schedule. Previous research by Ponnada et al. (34) showed increased participant non-response during periods of vigorous activity. Cauchard et al. (35) observed that running and cycling reduced accuracy in smartwatch vibrotactile pattern recognition compared to more moderate or sedentary activities. A typical duty day morning for military personnel might also include personal hygiene, breakfast, driving to work, preparing for training, reporting to duty, and morning meetings, which may have been contextual factors distracting from responding during the early morning window. Consideration of such activities may influence sampling strategy depending upon the characteristics of the constructs being assessed and anticipated contextual factors for non-response (i.e., scheduling sampling during time windows when an individual is most likely to respond). From a clinical perspective, the individual’s subjective description of constructs to be assessed should also guide the sampling strategy. For example, if a patient reports that their headaches typically occur upon waking early in the morning or late in the evening, then the windows for sampling should extend into or close to these timeframes to capture these data. Understanding how an individual’s daily routines and their observed symptom pattern may affect response behavior can enable the clinician and patient to co-create a sampling strategy that mitigates barriers to adherence and captures symptom data sufficiently.

#### 4.1.4 Cognitive survey completion

Mean participant cognitive survey completion was slightly less than the overall sample’s mean participant survey set completion. It is worth noting that the individuals in the 3-week study arm reported a range of cognitive challenges that could have affected their ability to respond to repeated assessments of their use of behavioral strategies throughout their day. Cognitive completion remained consistent across all 3 weeks in the 3-week study arm suggesting that response behavior was manageable within the context of a range of cognitive difficulties and in conjunction with cognitive rehabilitation.

#### 4.1.5 Lowest required adherence rate

Although previous methods of calculating completion and compliance reliably measure two aspects of response behaviors that represent adherence (13, 21), it remains unknown what the lowest adherence rate would be that still provides clinical utility. Although the evaluation of clinical utility of miEMA is beyond the scope of this article, we began to address this question with a review of the individual miEMA survey set completion results for each of the 3-week study arm participants. The 3-week arm participants were concurrently undergoing cognitive rehabilitation and received Cognitive Surveys personalized to their cognitive difficulties and treatment goals. They followed up with their treating provider weekly to review their miEMA data. Of these participants, the lowest survey set completion rate was 61.4%. This participant still provided 419 responses across four miEMA surveys (Mood, Headache, Fatigue, and Cognitive Surveys) over 21 consecutive days. Specifically, his responses to the Cognitive Survey prompts were sufficient and consistent enough for his treating Speech-Language Pathologist to be able to adjust his weekly treatments and to co-create “just in time” treatment adaptations with the participant. As such, his miEMA data showed increased endorsement of his use of cognitive strategies over the 3-week period. It is worth noting that this participant reported that he gained insights to his symptoms and cognitive functions based on his miEMA data as well as clinical benefit from the adaptive treatment adjustments offered by his treating provider.

Optimal adherence rates may vary between individuals depending on constructs and symptom patterns when considering the clinical utility of miEMA. However, minimizing interruption burden without the loss of considerable data is a known challenge of the miEMA approach (36) and has implications in military populations that may already be prone to survey fatigue. Li and colleagues’ (36) analyses of previous EMA datasets found that using machine learning to skip select questions based on a prediction uncertainty threshold and capping the number of questions in a survey set may reduce interruption burden while mitigating information loss. Achieving a high completion rate would undoubtedly provide more data to consider. However, identifying a “sweet spot,” or a point of diminishing returns, as to the least possible amount of participant-generated miEMA data required to attain optimal clinical applicability may be worthwhile for various constructs, conditions, and settings in future studies.

### 4.2 Usability

Participants reported high usability scores on the MAUQ and SUS when rating satisfaction with and usability of the C-STAR app and smartwatch platform.

#### 4.2.1 Mobile health application usability questionnaire

The overall MAUQ mean item scores and those for the EOU subscale were excellent. The mean item scores for the SIA subscale were satisfactory. All individual item mean scores, except for one item, on the SIA subscale were 6.0 (out of 7) or higher, indicating high perceived usability for how information was arranged in the app. The single exception was the item, “Whenever I made a mistake using the app, I could recover easily and quickly.” The mean score for this item across the entire sample was 4.7 (out of 7). Ponnada and colleagues (13) included an “Undo” option in their miEMA app and reported a 4.2% Undo rate. Participants in their study found the “Undo” feature useful in the situation of accidentally tapping the smartwatch so they could correct their response (13). The C-STAR app did not include an Undo feature; however, participants could opt to change their response until they tapped the *“Submit”* button. The *“Submit”* button appeared on the smartwatch face simultaneously with the response options, rather than on a separate screen after a response was entered, to keep device interaction time within the 3 to 5 s microinteraction timeframe. This relatively lower mean item score indicates a specific area for future improvement of the C-STAR app.

#### 4.2.2 System usability scale

The overall mean SUS score, as well as the mean scores for the Usable and Learnable subscales, were high in this sample. The average SUS benchmark score is 68 for digital health apps (excluding physical activity apps), with higher SUS scores correlating with more frequent system (e.g., app, website) use (37). Therefore, the mean overall SUS score of 89.0 suggests that the service members in the current study are highly likely to use the C-STAR app and smartwatch system based on its usability.

### 4.3 Limitations and future directions

This is the first study to evaluate the feasibility and usability of miEMA using a smartwatch in military personnel with a history of TBI. However, this study has some methodological limitations that should be highlighted. Our sample was small and primarily limited to Army service members; therefore, results may not generalize to other groups. Sampling was scheduled to start at 8 a.m. and stop at 6 p.m., so it remains unknown how life events that typically occur outside those hours (e.g., interacting with children and/or partners, after-work activities and responsibilities, extended work hours) may affect response behavior in this population. Additionally, the trial of miEMA data collection was limited to two or three weeks, therefore, it remains unknown how adherence may be affected over longer periods of time in a military patient population.

Future miEMA-related studies should be conducted in other military-connected groups (e.g., Veterans and/or retirees), service branches, and various contextual settings, specifically in field or austere environments (e.g., during field training exercises or deployment). Longitudinal trials of longer duration should also be a focus of future miEMA research in military populations. Sampling strategies and prediction models using machine learning to determine effective methods to reduce interruption burden and minimize information loss, yet still provide acceptable clinical utility should be evaluated in future miEMA studies.

## 5 Conclusion

This study demonstrated that miEMA is a feasible method of obtaining temporally-dense real-time subjective experience data in a sample of military personnel with a history of TBI, including those with cognitive challenges. Adherence rates in this military sample were similar to those in civilian populations, despite the differences in routines and work environments between military personnel and civilians. The C-STAR app and smartwatch had high usability ratings indicating that the system was likely compatible with the real-world work and living environments of service members in the sample. Our findings support the feasibility of miEMA to assess multiple constructs observed in service members with TBI and other various conditions and across other applications in the Military Health System.

## Data Availability

The datasets presented in this article are not publicly available due to ethical and privacy restrictions, but may be made available by request to the corresponding author upon required DoD/DHA approval. Requests to access the datasets should be directed to KM, katrina.s.monti.ctr@health.mil.

## Author’s note

The views expressed in this manuscript are those of the authors and do not necessarily represent the official policy or position of the Defense Health Agency, Department of Defense, or any other U.S. government agency. This work was prepared under Contract HT0014-22-C-0016 with DHA Contracting Office (NM-CD) HT0014 and, therefore, is defined as U.S. Government work under Title 17 U.S.C.§101. Per Title 17 U.S.C.§105, copyright protection is not available for any work of the U.S. Government. For more information, please contact dha.TBICOEinfo@health.mil.
