# Supplementary material for: Feasibility and usability of microinteraction ecological momentary assessment using a smartwatch in military personnel with a history of traumatic brain injury
Source: Front Neurol. 2025 Apr 14;16:1564657. doi: 10.3389/fneur.2025.1564657 (PMC12036483; doi:10.3389/fneur.2025.1564657)
Supplement: Supplementary file 1 [file Supplementary_file_1.docx]

**Supplementary Materials**

**Microinteraction Ecological Momentary Assessment (miEMA) Surveys**

Characteristics of each miEMA survey are presented in **Table 1** of the manuscript.

***Mood Survey.*** All study participants received the Mood Survey. The Mood Survey consisted of two prompts assessing valence and arousal levels to provide a temporal profile of the participant’s mood to potentially observe relationships between mood and other symptoms. Valence was annotated as “mood” in the initial survey question. The initial question, *“My mood is?”*, asked the participant to rate their current mood on a scale of 1 (negative) to 8 (positive). The second question, *“My arousal level is?”*, asked the participant to rate their current arousal level on a scale of 1 (low) to 8 (high). During the Smartwatch Orientation, the participant was instructed to interpret “arousal” as meaning “energy level.” The initial Mood Survey question was scheduled and delivered four times daily (once during each of the four windows). If the participant did not respond to the initial question upon delivery, the retry mechanism was activated and the initial question was delivered a second time during the same window of time. Delivery of the second question in the Mood Survey was contingent upon whether the participant responded to the initial question.

***Alcohol Survey.*** The Alcohol Survey was an option for participants to complete if they wanted to monitor their daily alcohol use. This survey consisted of two prompts that measures use during the previous 24 hours and number of standard drinks consumed and was only scheduled during the first window of the day (early morning, 8:00-10:30 am). If a participant selected the Alcohol Survey, they were given the Alcohol Standard Drink Form handout during the Smartwatch Orientation illustrating standard drink measurements according to alcohol type and volume. The first prompt required a “yes” or “no” response to the statement, *“I drank alcohol yesterday.”* If the participant responded, “No,” then the follow-up prompt was not delivered. If they responded, “Yes,” then they received the second prompt, *“How many standard drinks did you drink yesterday?*” Response options for the second prompt were: *1-2, 3-4, 5-6, >6?* If the participant did not respond to the initial question upon delivery, the retry mechanism was activated and the initial prompt was delivered a second time during the same window. Delivery of the second question in the Alcohol Survey was contingent upon whether the participant responded to the initial question. The second prompt did not have a retry mechanism.

***Self-efficacy Survey.*** Participants had the choice to complete the Self-efficacy Survey if tracking their ability to manage their symptoms was of interest to them. The Self-efficacy Survey was a single prompt personalized to the participant depending on what aspect of Self-efficacy the participant wanted to track. The purpose was to track the participant’s level of confidence in being able to manage different aspects of their life regarding their symptoms. For example, the participant would respond to the prompt, *“I can engage in daily activities,”* by rating their level of confidence to engage in daily activities on a scale of 1 (not confident) to 10 (very confident). The Self-efficacy Survey was a single prompt scheduled to be delivered once daily during the first window of the day (early morning, 8 am - 10:30 am). If the participant did not respond to the Self-efficacy prompt upon initial delivery, then the retry mechanism was activated and the question was delivered a second time during the same window.

***Headache Survey.*** Participants were given the option to complete the Headache Survey if headaches were a concerning symptom for them. The Headache Survey consisted of three questions assessing the participant’s frequency and severity of their headaches and interference of their headaches with daily activities. The first prompt required a “yes” or “no” response to the statement, *“I have a headache right now.”* If the participant responded, “No,” then the follow-up prompts were not delivered. If they responded, “Yes,” then they received the second prompt, *“My headache is…,”* asking them to rate their current headache severity on a scale of 1 (very mild) to 10 (very severe). If the participant did not respond to the second prompt, then the third prompt was not delivered. If the participant responded to the second prompt, then they would receive the third prompt, *“Headache is interfering with my activities?,”* asking them to choose a “yes” or “no” response. The initial prompt of the Headache Survey was scheduled and delivered four times a day (once during each of the four windows). If the participant did not respond to the initial question upon delivery, the retry mechanism was activated and the initial question was delivered a second time during the same window. Delivery of the second prompt was contingent upon a “yes” response endorsing the presence of a headache during the initial prompt. Delivery of the third prompt was contingent upon whether they entered a response for the second prompt. The second and third prompts of the Headache Survey did not have retry mechanisms.

***Fatigue Survey.*** Participants were offered the Fatigue Survey to complete if fatigue was a symptom of concern. The Fatigue Survey was structured similarly to the Headache Survey with a goal of understanding the frequency and severity of the participant’s fatigue and interference of fatigue in their daily activities. The initial prompt required a “yes” or “no” response to the statement, *“I feel fatigued right now.”* If the participant responded, “No,” then the follow-up prompts were not delivered. If they responded, “Yes,” then they would receive the second prompt, *“My fatigue is…,”* which required them to rate their fatigue severity on a scale of 1 (very mild) to 10 (very severe). If the participant responded to the second prompt, then they received the third prompt, *“Fatigue is interfering with my activities?,”* requiring them to choose a “yes” or “no” response. The initial prompt for the Fatigue Survey was scheduled and delivered four times daily (once during each of the four windows). If the participant did not respond to the initial question upon delivery, the retry mechanism was activated and the initial question was delivered a second time during the same window. Delivery of the second prompt was contingent upon a “yes” response endorsing fatigue during the initial prompt. Delivery of the third prompt was contingent upon whether they entered a response for the second prompt. The second and third prompts of the Fatigue Survey did not have retry mechanisms.

***Pain Survey.*** Participants were given the option to complete the Pain Survey if pain was a symptom of concern for them. The Pain Survey was structured similarly to the Headache and Fatigue Surveys with the aim of assessing the frequency and severity of pain and the interference of the individual’s pain in their daily activities. The initial prompt required a “yes” or “no” response to the statement, *“I am in pain right now.”* If the participant responded, “No,” then the follow-up prompts were not delivered. If they responded, “Yes,” then they received the second prompt, *“My pain is…”* which asked them to rate their pain severity on a scale of 1 (very mild) to 10 (very severe). If the participant responded to the second prompt, then they received the third prompt. The third prompt asked, *“Pain is interfering with my activities?”* with “yes” or “no” response options. The initial prompt in the Pain Survey was scheduled and delivered four times daily (once during each of the four windows). If the participant did not respond to the initial question upon delivery, the retry mechanism was activated and the initial question was delivered a second time during the same window. Delivery of the second prompt was contingent upon a “yes” response endorsing fatigued during the initial prompt. Delivery of the third prompt was contingent upon whether they entered a response for the second prompt. The second and third prompts of the Pain Survey did not have retry mechanisms.

***Cognitive Survey.*** All 3-week study arm participants completed Cognitive Surveys. The Cognitive Survey prompts were individualized to the participant’s treatment goals as part of their cognitive rehabilitation that they were concurrently receiving. The Cognitive Survey prompts were selected by the provider and participant from a 120-item statement bank. The Cognitive Survey prompts required a “yes” or “no” response to statements that described the participant’s adherence to cognitive behavioral strategies. Examples of statements are, *“I am adjusting when tasks become more challenging,”* and *“I am managing my time.”* The two Cognitive Survey prompts were scheduled as if they were individual surveys and delivered four times daily (once during each of the four windows). The delivery of the second Cognitive Survey prompt was not contingent upon a response to the first Cognitive Survey prompt. A retry mechanism was not implemented for the Cognitive Survey prompts, so if the participant did not respond to the Cognitive Survey prompt, then it was counted as a missed response.

**Pre-Trial Questionnaires**

***Ohio State University Traumatic Brain Injury (TBI) Identification Method (OSU-TBI ID).*** The OSU-TBI-ID is a standardized semi-structured face-to-face interview that ascertains by self-report a lifetime history of TBI including mechanism of injury, timing, and post-TBI symptoms (1). Research staff who were trained to give the interview would ask participants a series of questions related to potential mechanisms of injury (i.e., “Have you ever been involved in a motor vehicle accident in which you suffered a blow to your head or neck?”). Participants are then asked to estimate the age they were when the injury occurred and what symptoms were experienced such as feeling dazed, having a memory gap, experiencing loss of consciousness (LOC), and the LOC duration. Participants are also prompted to share periods of prolonged exposure to events with potential for TBI injury such as combat deployments. The OSU-TBI-ID allows the interviewer to determine a score of the worst injury experienced (1-5; a score of 1 meaning no history of TBI due to not reporting any injuries related to questions, never being dazed, not having a memory gap, or no LOC; a score of 5 indicating a severe TBI if the most severe injury reported involved LOC for greater than 24 hours). Interrater reliability was found to be high, r = 0.91 (1). Completion time typically ranged between 10-15 minutes depending on the comprehensiveness of the participant’s TBI history. Completion time: ~10-45 minutes.

***Neurobehavioral Symptom Inventory (NSI)*.** The NSI is a validated self-report measure aimed at assessing a range of neurobehavioral symptoms (e.g., somatosensory, affective, cognitive, and vestibular) after mild TBI (2). The NSI has demonstrated strong internal consistency with an alpha in the range of .88 to .92 (3). Items are rated on a 5-point Likert scale ranging from 0 (none) to 4 (very severe). A total score is calculated by the sum of the 22 items. Each of the subscale scores are also calculated by the sum of the items for the subscale. The NSI has demonstrated strong internal consistency for the total score (alpha=.95) and subscale scores also has shown good internal consistency with a coefficient alpha ranging from .88 to .92 (3). Completion time: ~5 minutes.

**Post-Trial Usability Questionnaires**

***Mobile Health Application Usability Questionnaire (MAUQ).*** The modified version of the MAUQ used in the current study is presented in **Table S1**. The MAUQ was developed to assess patients’ and providers’ perceived usability of mobile health (mHealth) apps (4,5). The original full measure is comprised of 21 items organized under three subscales (Ease of Use and Satisfaction (EOU), 8 items; System Information Arrangement (SIA), 6 items; and Usefulness, 7 items). However, we used only 12 items belonging to two subscales (EOU, 8 items; SIA, 4 items) in the current study with questions from the individual subscales intermixed throughout the survey. We opted to exclude two items from the original SIA subscale due to their irrelevance to the current study. The item, “This mHealth app provided an acceptable way to receive health care services,” was excluded because the app did not provide a way to receive health care services. The item, “This app has all the functions and capabilities I expect it to have,” was excluded because the app only served the research purposes of delivering survey prompts, collecting response data, and recording missed responses. The Usefulness subscale was not utilized because usefulness for health care was not an evaluated outcome in the current study. Participants were asked to rate each item on a scale from 1 (strongly disagree) to 7 (strongly agree). Item scores for the MAUQ and each subscale are reported in means and standard deviations with scores closer to 7 indicating stronger agreement with the item. Internal consistency for both the EOU and the SIA subscales was strong with Cronbach alpha values of .895 and .829, respectively (5). The MAUQ and its subscales have been correlated with two other commonly used usability questionnaires, the System Usability Scale (SUS) and the Post-Study System Usability Questionnaire (PSSUQ). The overall MAUQ has strong internal consistency (Cronbach alpha=.932) and correlation with the SUS, *r*=.6425 (5). The EOU subscale, which represents perceived interface quality, is strongly corelated with the third subscale of the PSSUQ (*r*=.8077). The SIA subscale, which indicates perceived system quality, is strongly correlated with the first subscale of the PSSUQ, *r*=0.7124 (5). Participants completed the MAUQ upon return of the smartwatch at their end of their trial and before any debriefing. Completion time: ~5 minutes.

***System Usability Scale (SUS).*** The SUS is a 10-item measure used to investigate human factors associated with interfacing with new information technology (6). The response options range from 1 (strongly disagree) to 5 (strongly agree). The statements alternate between positive and negative tones requiring reverse scoring of some items. The SUS was not modified for the current study. We used the previously described standard score conversion procedure to convert the raw scores (range, 0 – 40) to scores that range from 0 to 100 (7). We then calculated a percentile from the converted score. Results are reported in mean total converted scores, percentiles, and standard deviations. The average SUS benchmark score is 68 for digital health apps (excluding physical activity apps), with higher SUS scores correlating with more frequent system (e.g., app, website) use (8). Internal consistency for the SUS has been reasonable to strong with Cronbach’s alpha ranging from .70 to .95 (9). We also calculated scores for two factors – Usable (8 items) and Learnable (2 items) – potentially providing more distinct insight to the perceived usability and learnability of the app and smartwatch (10). The Usable subscale and overall SUS score are highly correlated, *r*=.985 (10). The coefficient alpha for each of the Usable and Learnable subscales were .91 and .70, respectively, indicating good to strong reliability of each subscale (10). The Usable subscale score was calculated by multiplying the summed score contributions of SUS items 1 - 3 and 5 - 9 by 3.125. The Learnable subscale score was calculated by multiplying the summed score contributions of SUS items 4 and 10 by 12.5. The procedure to calculate the Usable and Learnable subscales has been described previously by Sauro and Lewis (7). Participants completed the SUS upon return of the smartwatch at the end of their trial and before their feedback session. Completion time: ~3-5 minutes.

**TABLE S1.** Mobile Health Application Usability Questionnaire (MAUQ)

| **1.The app was easy to use.** | | | | | | |
| --- | --- | --- | --- | --- | --- | --- |
| Strongly Disagree |  |  | Neither Disagree or Agree |  |  | Strongly Agree |
| 1 | 2 | 3 | 4 | 5 | 6 | 7 |
| **2. It was easy for me to learn to use the app.** | | | | | | |
| 1 | 2 | 3 | 4 | 5 | 6 | 7 |
| **3. The navigation was consistent when moving between screens.** | | | | | | |
| 1 | 2 | 3 | 4 | 5 | 6 | 7 |
| **4. The interface of the app allowed me to use all the functions (such as entering information, responding to reminders, viewing information) offered by the app.** | | | | | | |
| 1 | 2 | 3 | 4 | 5 | 6 | 7 |
| **5. Whenever I made a mistake using the app, I could recover easily and quickly.** | | | | | | |
| 1 | 2 | 3 | 4 | 5 | 6 | 7 |
| **6. I like the interface of the app.** | | | | | | |
| 1 | 2 | 3 | 4 | 5 | 6 | 7 |
| **7. The information in the app was well organized, so I could easily find the information I needed.** | | | | | | |
| 1 | 2 | 3 | 4 | 5 | 6 | 7 |
| **8. The app adequately acknowledged and provided information to let me know the progress of my action.** | | | | | | |
| 1 | 2 | 3 | 4 | 5 | 6 | 7 |
| **9. I feel comfortable using the app in social settings.** | | | | | | |
| 1 | 2 | 3 | 4 | 5 | 6 | 7 |
| **10. The amount of time involved in using this app has been fitting for me.** | | | | | | |
| 1 | 2 | 3 | 4 | 5 | 6 | 7 |
| **11. I would use this app again.** | | | | | | |
| 1 | 2 | 3 | 4 | 5 | 6 | 7 |
| **12. Overall, I am satisfied with this app.** | | | | | | |
| 1 | 2 | 3 | 4 | 5 | 6 | 7 |

**References**

1. Corrigan JD, Bogner J. Initial reliability and validity of the Ohio State University TBI Identification Method. J Head Trauma Rehabil. 2007;22(6):318–29.

2. Meterko M, Baker E, Stolzmann KL, Hendricks AM, Cicerone KD, Lew HL. Psychometric assessment of the Neurobehavioral Symptom Inventory-22: the structure of persistent postconcussive symptoms following deployment-related mild traumatic brain injury among veterans. The Journal of Head Trauma Rehabilitation. 2012;27(1):55–62.

3. King PR, Donnelly KT, Donnelly JP, Dunnam M, Warner G, Kittleson CJ, Bradshaw CB, Alt M, Meier ST. Psychometric study of the Neurobehavioral Symptom Inventory. J Rehabil Res Dev. 2012;49(6):879–88.

4. Kortum P, Sorber M. Measuring the usability of mobile applications for phones and tablets. International Journal of Human-Computer Interaction (Internet). 2015 Aug 3 (cited 2024 Dec 5);31:518–29. Available from: https://www.researchgate.net/publication/281717032_Measuring_the_Usability_of_Mobile_Applications_for_Phones_and_Tablets

5. Zhou L, Bao J, Setiawan IMA, Saptono A, Parmanto B. The mHealth App Usability Questionnaire (MAUQ): development and validation study. JMIR mHealth and uHealth (Internet). 2019 Apr 11 (cited 2024 Feb 29);7(4):e11500. Available from: https://mhealth.jmir.org/2019/4/e11500

6. Brooke J. SUS: A “quick and dirty” usability scale. In: Usability Evaluation in Industry (Internet). London, UK: Taylor & Francis; 1996 (cited 2024 Feb 29). p. 189–94. Available from: https://www.researchgate.net/publication/319394819_SUS_--_a_quick_and_dirty_usability_scale

7. Sauro J, Lewis JR. Standardized usability questionnaires. In: Quantifying the User Experience: Practical Statistics for User Research (Internet). 2nd ed. Cambridge, MA: Morgan Kaufmann; 2016 (cited 2024 Dec 5). p. 198–210. Available from: https://www.sciencedirect.com/book/9780123849687/quantifying-the-user-experience

8. Hyzy M, Bond R, Mulvenna M, Bai L, Dix A, Leigh S, Hunt S. System Usability Scale benchmarking for digital health apps: meta-analysis. JMIR mHealth and uHealth (Internet). 2022 Aug 18 (cited 2024 Dec 5);10(8):e37290. Available from: https://mhealth.jmir.org/2022/8/e37290

9. Lewis JR. The System Usability Scale: past, present, and future. International Journal of Human–Computer Interaction (Internet). 2018 Jul 3 (cited 2024 Jul 10);34(7):577–90. Available from: https://doi.org/10.1080/10447318.2018.1455307

10. Lewis JR, Sauro J. The factor structure of the System Usability Scale. In: Kurosu M, editor. Human Centered Design (Internet). Berlin, Heidelberg: Springer Berlin Heidelberg; 2009 (cited 2024 Dec 5). p. 94–103. Available from: https://doi.org/10.1007/978-3-642-02806-9_12
